# Supplementary material for: Effective Absorption of Dichloromethane Using Carboxyl-Functionalized Ionic Liquids
Source: Int J Environ Res Public Health. 2023 May 11;20(10):5787. doi: 10.3390/ijerph20105787 (PMC10218073; doi:10.3390/ijerph20105787)
Supplement: Supplementary file 1 [file ijerph-20-05787-s001.zip › ijerph-2269389-supplementary.pdf]

Supporting Information

Effective absorption of dichloromethane using carboxyl-functionalized ionic liquids

Mengjun Wang,[1,2] Manman Zhang,[2] Shaojuan Zeng,[3] Yi Nie,[2,3] Tao Li,[1] Baozeng Ren,[1] Yinge Bai,[2,3,4*] Xiangping Zhang[2,3,5*]

[1] College of Chemical and Engineering, Zhengzhou University, Zhengzhou 450001, China

[2] Longzihu New Energy Laboratory, Zhengzhou Institute of Emerging Industrial Technology, Zhengzhou 450000, China

[3] CAS Key Laboratory of Green Process and Engineering, State Key Laboratory of Multiphase Complex Systems, Beijing Key Laboratory of Ionic Liquids Clean Process, Institute of Process Engineering, Chinese Academy of Sciences, Beijing 100190, China

[4] Langfang Green Industrial Technology Center, Langfang 065000, China

[5] College of Chemical Engineering and Environment, China University of Petroleum, Beijing 102249, China

Section S1. Characterization of ILs

S1.1. The FT-IR spectrum and ¹H-NMR of ILs

(1) [N1888][Ac]

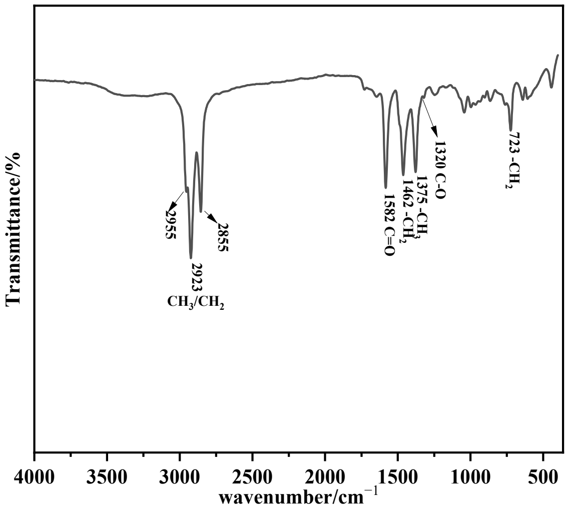

Figure S1. The FT-IR spectrum of [N1888][Ac].

The ¹H-NMR (d6-DMSO) spectrum of [N1888][Ac] is shown in Figure S2: 3.20[t, 6H], 2.95[s, 3H], 1.60[s, 6H], 1.52[s, 3H], 1.27[m, 30H], 0.87[t, 9H].

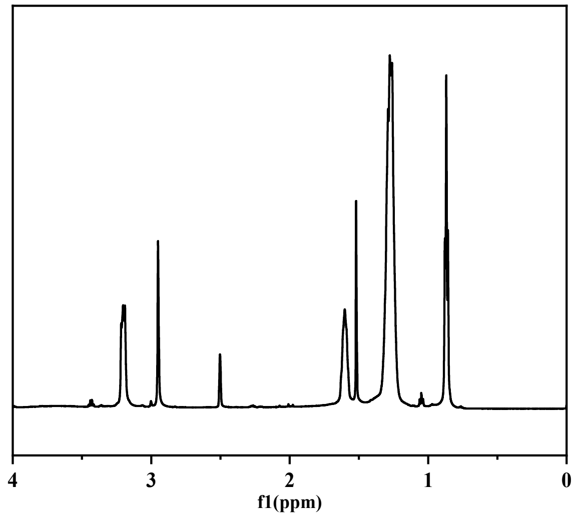

Figure S2. The ¹H-NMR spectrum of [N1888][Ac].

(2) [N_{1888}][FA]

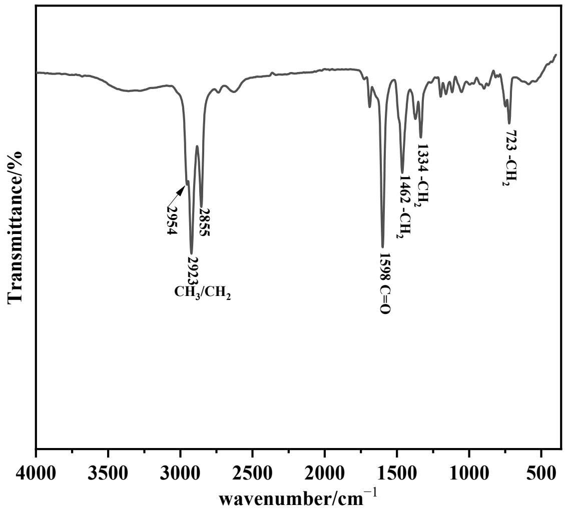

Figure S3. The FT-IR spectrum of [N_{1888}][FA].

The ^{1}H-NMR (d_6-DMSO) spectrum of [N_{1888}][FA] is shown in Figure S4: 3.35[s, 1H], 3.20[s, 6H], 2.93[m, 3H], 1.61[s, 6H], 1.29[s, 30H], 0.87[t, 9H].

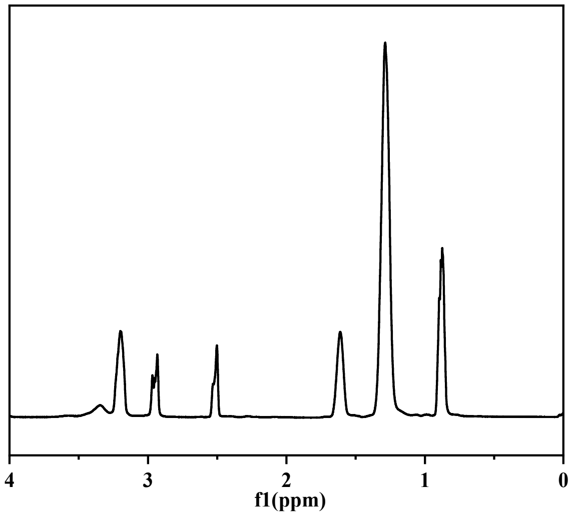

Figure S4. The ^{1}H-NMR spectrum of [N_{1888}][FA].

(3) [N1888][Gly]

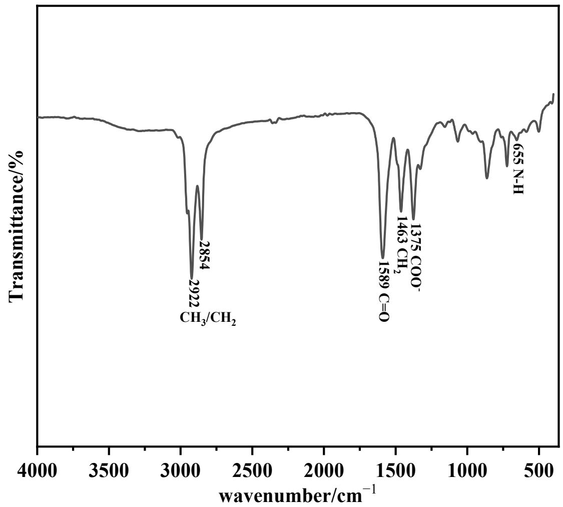

Figure S5. The FT-IR spectrum of [N1888][Gly].

The ^{1}H-NMR (d6-DMSO) spectrum of [N1888][Gly] is shown in Figure S6: 3.18[m, 6H], 2.98[s, 2H], 2.93[s, 3H], 1.60[m, 6H], 1.26[m, 30H], 0.87[m, 9H].

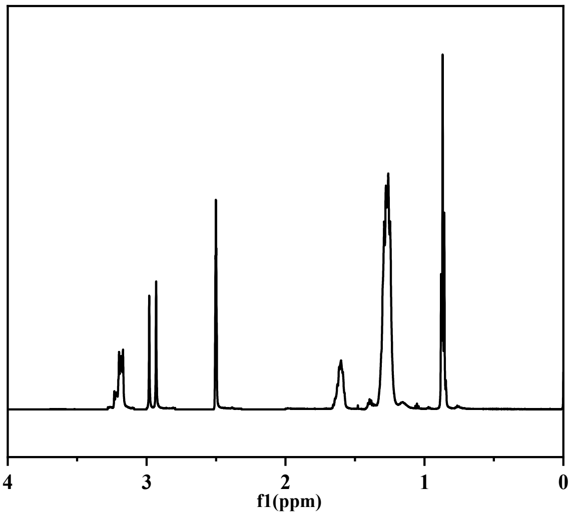

Figure S6. The ^{1}H-NMR spectrum of [N1888][Gly].

(4) [P_{66614}][Gly]

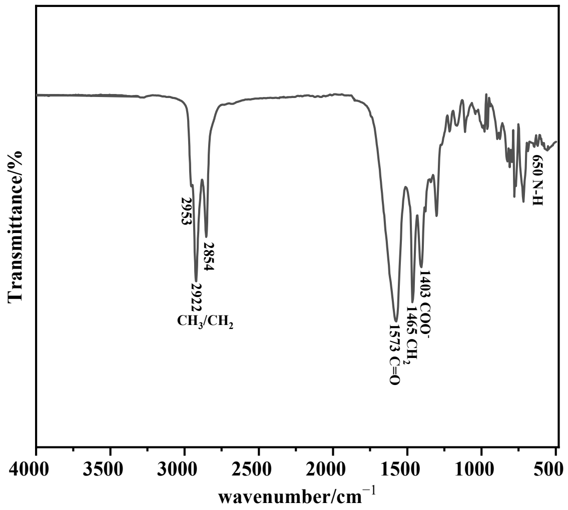

Figure S7. The FT-IR spectrum of [P_{66614}][Gly].

The ^{1}H-NMR (d_6-DMSO) spectrum of [P_{66614}][Gly] is shown in Figure S8: 3.34[s, 2H], 2.17[m, 8H], 1.24-1.46[m, 48H], 0.87[m, 12H].

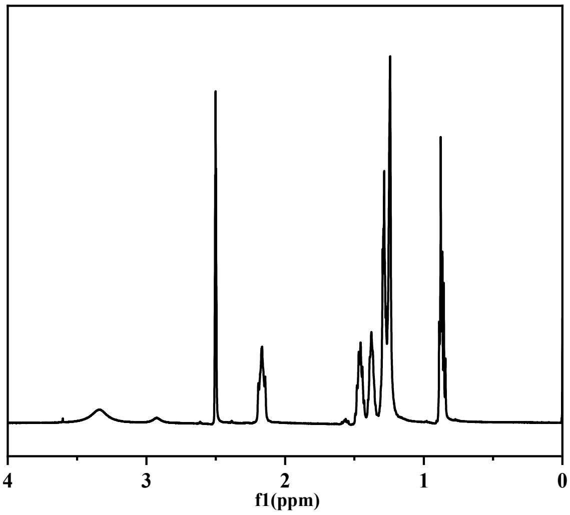

Figure S8. The ^{1}H-NMR spectrum of [P_{66614}][Gly].

S1.2. Density and viscosity at different temperatures of four ILs

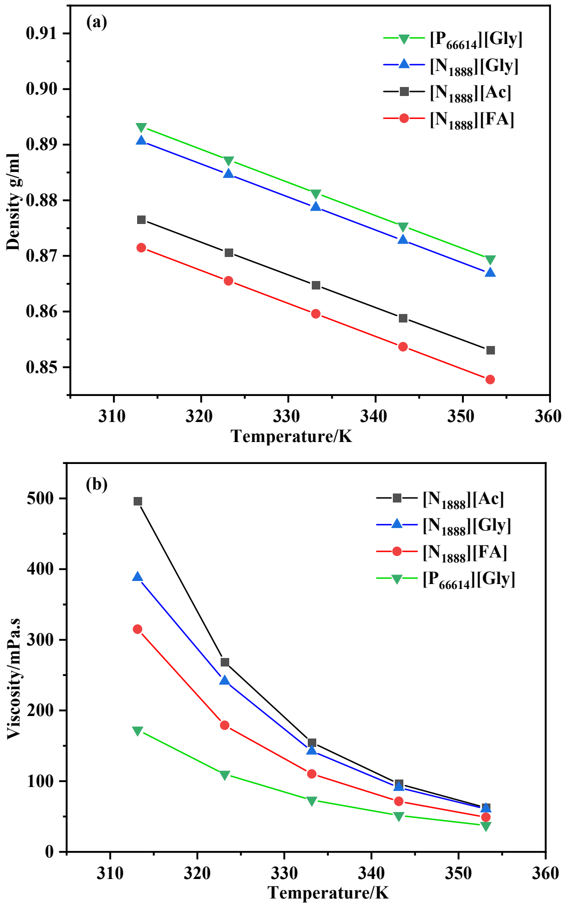

Figure S9. The density (a) and viscosity (b) at different temperatures of ILs.

Table S1. Density and viscosity of four ILs at 313.15 K.

ILs	Density(g/ml)	Viscosity(mPa·s)
$[N_{1888}][Ac]$	0.8765	496.3
$[N_{1888}][FA]$	0.8714	315.1
$[N_{1888}][Gly]$	0.8906	388.1
$[P_{66614}][Gly]$	0.8932	172.26

S1.3. The thermogravimetric analysis of ILs

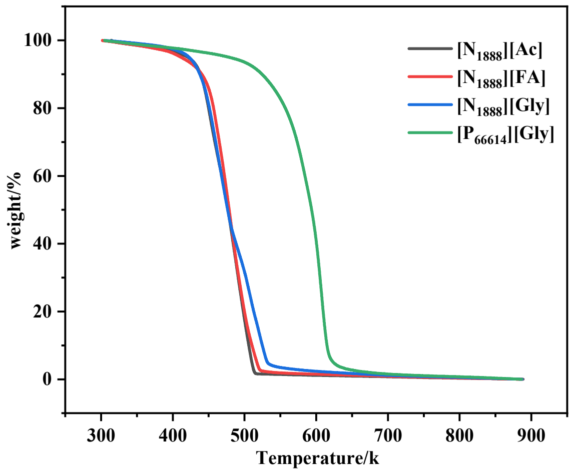

Figure S10. Thermal gravity analysis of four ILs.

Table S2. Thermal Properties of ILs.

IL	T_d^*/K
[N_{1888}][Ac]	440.88
[N_{1888}][FA]	445.50
[N_{1888}][Gly]	435.45
[P_{66614}][Gly]	556.58

T_d^* is defined as the temperature at which the ionic liquid loses 5% of its initial mass.

Section S2. Apparatus and procedure

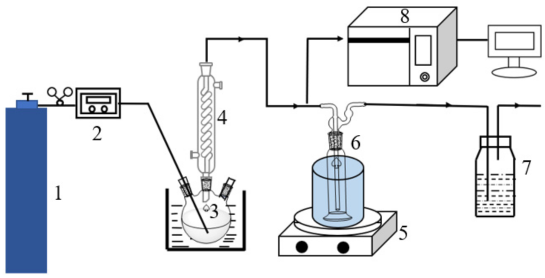

Figure S11. Experimental device for IL to absorb DCM containing vapor: (1) N_2 tank; (2) mass flow controller; (3) three-necked flask; (4) serpentine condenser; (5) magnetic heating stirrer; (6) absorption bottle; (7) exhaust gas absorption bottle; (8) gas chromatograph.

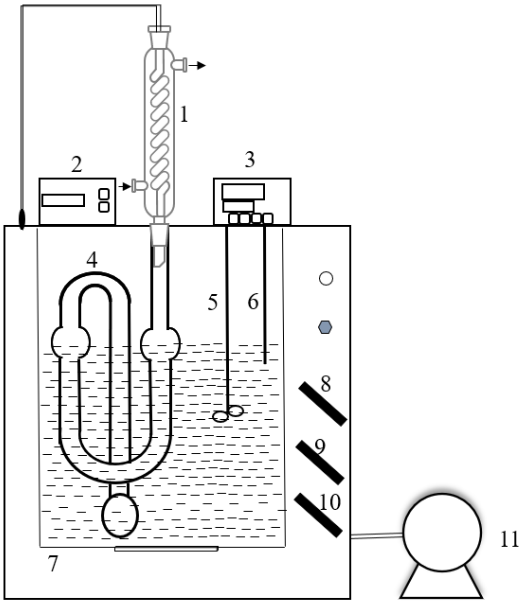

Figure S12. Diagram of the VLE measurement experiment: (1) serpentine condenser; (2) pressure indicator; (3) temperature indicator; (4) u-balance tube; (5) stirrer; (6) thermometer; (7) constant temperature water bath; (8) atmospheric relief valve; (9) buffer gas tank valve; (10) vacuum pump valve; (11) vacuum pump.

Section S3. NRTL model

The influence of ILs on the vapor pressure of DCM can be expressed by the activity coefficient, which can be calculated by the phase equilibrium Eq.(1)[1]:

$$\gamma_i = \frac{y_i \hat{\Phi}_i P}{x_i \Phi_i^s P_i^s} \tag{1}$$

where P and p_i^s are the vapor pressures of the binary system and pure component i at the system temperature, respectively, and p_i^s can be calculated by the Antoine equation[2]. y_i and x_i are the mole fraction of component i in the vapor phase and liquid phase, respectively. $\hat{\Phi}_i$ is the fugacity coefficient of solvent i in the vapor mixture, and Φ_i^s is the fugacity coefficient of pure component i in its saturated state.

For an IL-containing binary system, solvent (1) + IL (2), the vapor phase is pure solvent vapor due to the nonvolatility of ILs, and thus y_1=1. Considering that the vapor phase composition of the binary system and pure solvent is the same, and the pressure difference between them ($P - P_1^s$) is relatively small, the assumption of ideal behavior is adopted for the vapor at low pressure. Therefore, the fugacity coefficient ratio is approximately unity. Thus, Eq. (1) can be simplified as Eq. (2):

$$\gamma_1 = \frac{P}{P_1^s x_1} \tag{2}$$

References

1. Zhao, J.; Jiang, X.C.; Li, C. X.; Wang, Z. H. Vapor pressure measurement for binary and ternary systems containing a phosphoric ionic liquid. *FLUID PHASE EQUILIBR*. **2006,** *247* (1-2), 190-198.
2. Apelblat, A.; Wisniak, J.; Tamir, A. Vapor-liquid equilibriums in the dichloromethane-chlorobromomethane-dibromomethane system and its binaries. *J. Chem. Eng. Data*. **1981,** *26* (2), 144-147.

Section S4. Reliability of experimental equipwent

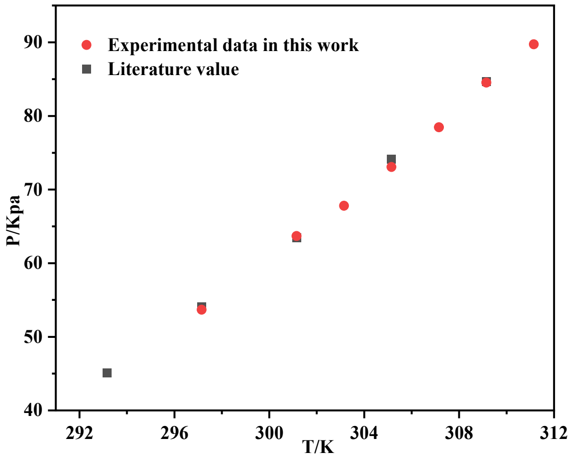

Figure S13. Vapor pressures of DCM (1) + [Emim][Ac] (2) (x_1=0.9) obtained in this work and literature values from Gui et al.[1]

References

1. Gui, C.; Li, G.; Zhu, R.; Lei, Z.; Dong, Y. Capturing VOCs in the pharmaceutical industry with ionic liquids. *Chem. Eng. Sci.* **2022,** *252*, 117504.

Section S5. The experimental reagents

Table S3. The details of the chemicals involved in this work.

Chemicals	Abbreviations	CAS	Purity	Manufacturer
Nitrogen	N_2	7727-37-9	>99.99%	Henan Yuanzheng Special Gas Co., Ltd
Trioctylmethylammonium chloride	$[N_{1888}][Cl]$	5137-55-3	97%	Shanghai Maclin Biochemical Technology Co., Ltd
Trihexyl(tetradecyl)phosphonium Chloride	$[P_{66614}][Cl]$	258864-54-9	97%	Jiangsu Aikang Biomedical Research and Development Co., Ltd
Anhydrous ethanol	C_2H_5OH	64-17-5	⩾99.7%	Wuxi Yatai United Chemical Co., Ltd
Dichloromethane	DCM	75-09-2	⩾99.5%	Shanghai Taitan Technology Co., Ltd
sodium acetate	$C_2H_3NaO_2$	127-09-3	99%	Shanghai Taitan Technology Co., Ltd
sodium formate	HCOONa	141-53-7	⩾98.5%	Shanghai Taitan Technology Co., Ltd
glycine	Glycine	56-40-6	99%	Shanghai Taitan Technology Co., Ltd

Section S6. The experimental and NRTL predicted values of vapor pressure for the DCM + ILs binary system at different temperatures and different molar compositions

Table S4. Experimental and NRTL model calculated vapor pressure values of DCM (1) + $[N_{1888}][Ac]$ (2) binary system at different temperatures and different molar compositions.

T/K	P^{exp}/kPa	x_1	P_1^s/kPa	P^{NRTL}/kPa	γ_1^{exp}	γ_1^{NRTL}	RD(P)
297.15	56.17	1	55.7032	55.70	1.0084	1.0000	0.0083
300.15	62.92	1	62.7521	62.75	1.0026	1.0000	0.0026
303.15	71.04	1	70.5000	70.50	1.0076	1.0000	0.00076
306.15	79.16	1	78.9955	78.96	1.0021	1.0000	0.0021
309.15	88.80	1	88.2892	88.29	1.0058	1.0000	0.0058
297.15	52.65	0.95	55.7032	52.88	0.9950	0.9993	0.0043
300.15	59.44	0.95	62.7521	59.57	0.9971	0.9992	0.0021
303.15	67.79	0.95	70.5000	66.92	1.0122	0.9992	0.0128
306.15	75.80	0.95	78.9955	74.98	1.0100	0.9992	0.0108
309.15	82.70	0.95	88.2892	83.80	0.9860	0.9991	0.013
297.15	49.74	0.9	55.7032	49.97	0.9922	0.9967	0.0045
300.15	56.22	0.9	62.7521	56.28	0.9956	0.9965	0.0010
303.15	62.62	0.9	70.5000	63.22	0.9869	0.9965	0.0097
306.15	69.45	0.9	78.9955	70.83	0.9768	0.99963	0.0196
309.15	78.02	0.9	88.2892	79.15	0.9819	0.9961	0.0145
297.15	48.45	0.85	55.7032	46.96	1.0232	0.9917	0.0308
300.15	53.40	0.85	62.7521	52.88	1.0011	0.99914	0.0097
303.15	59.39	0.85	70.5000	59.39	0.9911	0.9910	0.0000
306.15	66.09	0.85	78.9955	66.52	0.9843	0.9907	0.0064
309.15	74.51	0.85	88.2892	74.32	0.9928	0.9903	0.0025
297.15	44.11	0.8	55.7032	43.82	0.9898	0.9834	0.0064
300.15	49.77	0.8	62.7521	49.34	0.9915	0.9827	0.0088
303.15	55.40	0.8	70.5000	55.39	0.9823	0.9820	0.0003
306.15	61.20	0.8	78.9955	62.02	0.9684	0.9813	0.0013
309.15	69.64	0.8	88.2892	69.26	0.9860	0.9806	0.0055
						ARD	0.81%

Table S5. Experimental and NRTL model calculated vapor pressure values of DCM (1) + $[N_{1888}][FA]$ (2) binary system at different temperatures and different molar compositions.

T/K	P^{exp}/kPa	x_1	P_1^s/kPa	P^{NRTL}/kPa	γ_1^{exp}	γ_1^{NRTL}	RD(P)
297.15	56.17	1	55.7032	55.70	1.0084	1.0000	0.0083
300.15	62.92	1	62.7521	62.75	1.0026	1.0000	0.0026
303.15	71.04	1	70.5000	70.50	1.0076	1.0000	0.0076
306.15	79.16	1	78.9955	78.99	1.0021	1.0000	0.0021

309.15	88.80	1	88.2892	88.29	1.0058	1.0000	0.0058
297.15	51.93	0.95	55.7032	52.49	0.9813	0.9920	0.0109
300.15	58.07	0.95	62.7521	59.15	0.9741	0.9922	0.0186
303.15	66.91	0.95	70.5000	66.47	0.9991	0.9924	0.0066
306.15	74.58	0.95	78.9955	74.49	0.9938	0.9926	0.0011
309.15	81.80	0.95	88.2892	83.28	0.9753	0.9928	0.0180
297.15	48.97	0.9	55.7032	48.75	0.9768	0.9724	0.0045
300.15	53.65	0.9	62.7521	54.96	0.9500	0.9731	0.0244
303.15	60.75	0.9	70.5000	61.79	0.9574	0.9739	0.0172
306.15	68.12	0.9	78.9955	69.29	0.9582	0.9745	0.0171
309.15	76.18	0.9	88.2892	77.49	0.9587	0.9752	0.0172
297.15	45.78	0.85	55.7032	44.81	0.9668	0.9464	0.0016
300.15	50.04	0.85	62.7521	50.50	0.9381	0.9477	0.0103
303.15	57.74	0.85	70.5000	56.87	0.9636	0.9450	0.0151
306.15	64.28	0.85	78.9955	63.81	0.9573	0.9503	0.0073
309.15	71.19	0.85	88.2892	71.41	0.9486	0.9515	0.0031
297.15	42.45	0.8	55.7032	40.87	0.9526	0.9712	0.0371
300.15	46.08	0.8	62.7521	46.14	0.9178	0.9191	0.0014
303.15	52.13	0.8	70.5000	51.95	0.9242	0.9210	0.0034
306.15	58.40	0.8	78.9955	58.32	0.9240	0.9229	0.0012
309.15	65.20	0.8	88.2892	65.31	0.9231	0.9246	0.0016
						ARD	1.06%

Table S6. Experimental and NRTL model calculated vapor pressure values of DCM (1) + $[N_{1888}][Gly]$ (2) binary system at different temperatures and different molar compositions.

T/K	P^{exp}/kPa	x_1	P_1^s/kPa	P^{NRTL}/kPa	γ_1^{exp}	γ_1^{NRTL}	RD(P)
297.15	56.17	1	55.7032	55.70	1.0084	1.0000	0.0083
300.15	62.92	1	62.7521	62.75	1.0026	1.0000	0.0026
303.15	71.04	1	70.5000	70.50	1.0076	1.0000	0.0076
306.15	79.16	1	78.9955	78.99	1.0021	1.0000	0.0021
309.15	88.80	1	88.2892	88.29	1.0058	1.0000	0.0058
297.15	51.58	0.95	55.7032	51.98	0.9746	0.9822	0.0077
300.15	57.39	0.95	62.7521	58.59	0.9626	0.9827	0.0209
303.15	66.84	0.95	70.5000	65.86	0.9980	0.9833	0.0147
306.15	74.31	0.95	78.9955	73.83	0.9902	0.9838	0.0064
309.15	82.33	0.95	88.2892	82.56	0.9816	0.9843	0.0028
297.15	46.23	0.9	55.7032	47.26	0.9221	0.9427	0.0223
300.15	52.89	0.9	62.7521	53.33	0.9365	0.9444	0.0084
303.15	59.94	0.9	70.5000	60.02	0.9447	0.9460	0.0014
306.15	66.28	0.9	78.9955	67.36	0.9323	0.9475	0.0164
309.15	75.33	0.9	88.2892	75.41	0.9480	0.9490	0.0010
297.15	41.64	0.85	55.7032	42.38	0.8794	0.8951	0.0179
300.15	46.85	0.85	62.7521	47.89	0.8784	0.8979	0.0222

303.15	53.58	0.85	70.5000	53.97	0.8941	0.9005	0.0072
306.15	60.80	0.85	78.9955	60.64	0.9055	0.9031	0.0026
309.15	66.41	0.85	88.2892	67.96	0.8849	0.9056	0.0234
297.15	38.88	0.8	55.7032	37.72	0.8725	0.8464	0.0300
300.15	44.17	0.8	62.7521	42.68	0.8859	0.8501	0.0404
303.15	48.55	0.8	70.5000	48.15	0.8607	0.8536	0.0083
306.15	55.39	0.8	78.9955	54.17	0.8765	0.8571	0.0221
309.15	60.69	0.8	88.2892	60.77	0.8592	0.8604	0.0014
						ARD	1.22%

Table S7. Experimental and NRTL model calculated vapor pressure values of DCM (1) + [P_{66614}][Gly] (2) binary system at differenat temperatures and different molar compositions.

T/K	P^{exp}/kPa	x_1	P_1^s/kPa	P^{NRTL}/kPa	γ_1^{exp}	γ_1^{NRTL}	RD(P)
297.15	56.17	1	55.7032	55.70	1.0084	1.0000	0.0083
300.15	62.92	1	62.7521	62.75	1.0026	1.0000	0.0026
303.15	71.04	1	70.5000	70.50	1.0076	1.0000	0.0076
306.15	79.16	1	78.9955	78.99	1.0021	1.0000	0.0021
309.15	88.80	1	88.2892	88.29	1.0058	1.0000	0.0058
297.15	50.50	0.95	55.7032	51.03	0.9543	0.9642	0.0104
300.15	56.78	0.95	62.7521	57.56	0.9524	0.9655	0.0137
303.15	64.11	0.95	70.5000	64.74	0.9572	0.9667	0.0099
306.15	71.56	0.95	78.9955	72.63	0.9535	0.9678	0.0150
309.15	79.23	0.95	88.2892	81.27	0.9446	0.9689	0.0257
297.15	43.60	0.9	55.7032	44.82	0.8697	0.8940	0.0280
300.15	49.02	0.9	62.7521	50.67	0.8680	0.8972	0.0338
303.15	55.97	0.9	70.5000	57.13	0.8821	0.9003	0.0207
306.15	63.71	0.9	78.9955	64.22	0.8962	0.9033	0.0080
309.15	70.63	0.9	88.2892	72.01	0.8889	0.9062	0.0194
297.15	38.02	0.85	55.7032	38.78	0.8030	0.8191	0.0201
300.15	43.19	0.85	62.7521	43.95	0.8097	0.8239	0.0176
303.15	49.16	0.85	70.5000	49.65	0.8195	0.8285	0.0096
306.15	56.41	0.85	78.9955	55.93	0.8402	0.8330	0.0085
309.15	63.73	0.85	88.2892	62.84	0.8492	0.8373	0.0140
297.15	33.10	0.8	55.7032	33.42	0.7428	0.7499	0.0096
300.15	38.63	0.8	62.7521	37.94	0.7696	0.7558	0.0178
303.15	43.27	0.8	70.5000	42.95	0.7673	0.7615	0.0075
306.15	48.85	0.8	78.9955	48.47	0.7730	0.7670	0.0077
309.15	57.89	0.8	88.2892	54.55	0.8195	0.7723	0.0576
						ARD	1.53%

Section S7. The structures of DCM and IL pairs

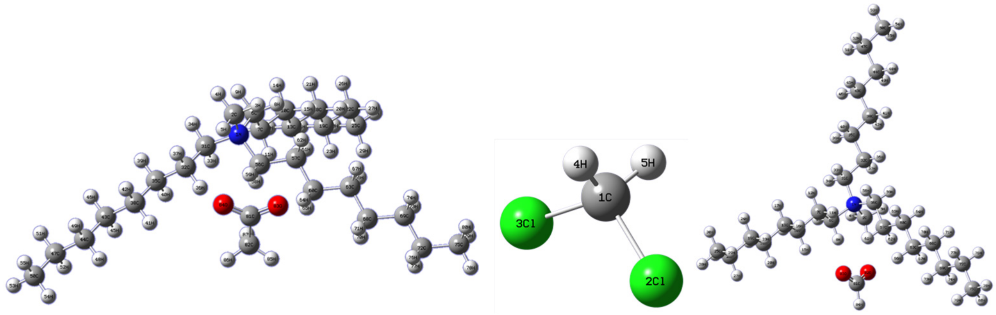

(a) $[N_{1888}][Ac]$ (b) DCM (c) $[N_{1888}][FA]$

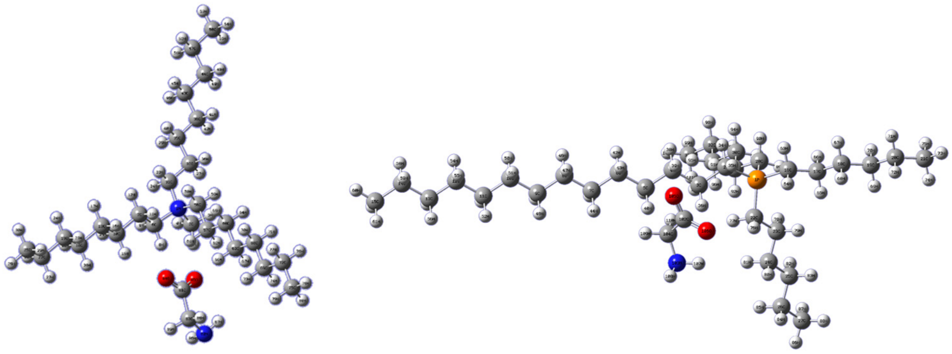

(d) $[N_{1888}][Gly]$ (e) $[P_{66614}][Gly]$

Figure S14. The structures of DCM and IL pairs.

Section S8. Optimized structures of DCM-ion system

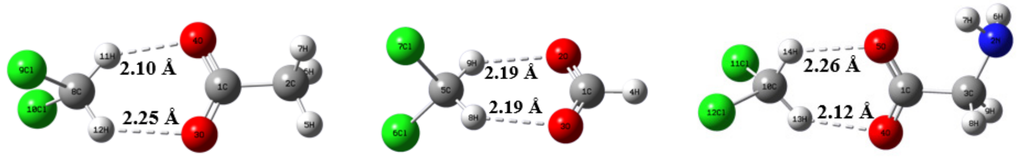

(a) $[Ac]^{-}$-DCM (b) $[FA]^{-}$-DCM (c) $[Gly]^{-}$-DCM

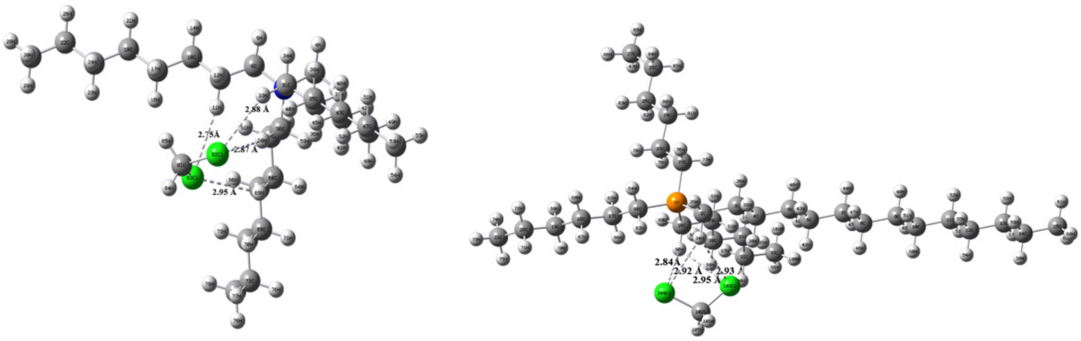

(d) $[N_{1888}]^{+}$-DCM (e) $[P_{66614}]^{+}$-DCM

Figure 15. Optimized structures of DCM-ion system.

Section S9. The atomic numbering, molecular coordinates of DCM, ILs and ILs + DCM

Table S8. The Atomic Numbering, Molecular Coordinates of [N_{1888}][Ac] + DCM.

atomic numbering	atom	coordinate		
1	N	-0.021111	-0.402414	2.579436
2	C	0.272756	0.086827	3.962871
3	H	-0.58266	0.64433	4.338598
4	H	0.467714	-0.76901	4.609064
5	H	1.146481	0.733224	3.936073
6	C	-1.284561	-1.252999	2.64099
7	C	-1.777066	-1.792193	1.304406
8	H	-2.048817	-0.629706	3.107534
9	H	-1.059313	-2.070084	3.331315
10	C	-3.107845	-2.533719	1.475763
11	H	-1.903529	-0.979036	0.587097
12	H	-1.036302	-2.462362	0.86517
13	C	-3.623553	-3.093467	0.145896
14	H	-2.993243	-3.354421	2.196303
15	H	-3.860117	-1.854515	1.898468
16	H	-3.720094	-2.272713	-0.574614
17	H	-2.872198	-3.774672	-0.271714
18	C	-4.96176	-3.825982	0.272199
19	C	-5.470266	-4.387415	-1.059045
20	H	-5.713339	-3.141543	0.68714
21	H	-4.862372	-4.645133	0.996629
22	C	-6.810826	-5.119669	-0.941688
23	H	-5.566908	-3.568867	-1.783782
24	H	-4.719142	-5.071869	-1.474033
25	C	-7.308073	-5.676312	-2.278783
26	H	-6.712653	-5.937543	-0.217155
27	H	-7.560678	-4.434289	-0.527689
28	H	-8.265251	-6.1935	-2.167888
29	H	-7.444653	-4.874754	-3.011625
30	H	-6.590692	-6.388285	-2.699182
31	C	1.129747	-1.281466	2.073571
32	C	2.507863	-0.627133	2.06307
33	H	0.864703	-1.550171	1.048089
34	H	1.124477	-2.17252	2.706951
35	C	3.470091	-1.459909	1.200805
36	H	2.452556	0.372544	1.628531
37	H	2.914541	-0.517036	3.074475
38	C	4.827222	-0.775124	1.017991
39	H	3.610115	-2.451956	1.649049

40	H	3.006978	-1.624618	0.222648
41	H	4.665168	0.231367	0.615245
42	H	5.315526	-0.644393	1.993072
43	C	5.757972	-1.539952	0.07382
44	C	7.08164	-0.815091	-0.183613
45	H	5.241363	-1.694906	-0.880456
46	H	5.959269	-2.538644	0.483461
47	C	8.011429	-1.565668	-1.142171
48	H	6.868899	0.180669	-0.592975
49	H	7.601444	-0.649508	0.769734
50	C	9.327083	-0.826671	-1.402749
51	H	8.223713	-2.561746	-0.734238
52	H	7.488706	-1.730606	-2.092006
53	H	9.9714	-1.381806	-2.090488
54	H	9.142676	0.159413	-1.840894
55	H	9.884273	-0.675164	-0.472394
56	C	-0.191238	0.783489	1.62266
57	C	-1.445831	1.627016	1.807385
58	H	-0.173603	0.381023	0.606541
59	H	0.69274	1.402103	1.758985
60	C	-1.44016	2.749131	0.756674
61	H	-2.345004	1.023188	1.657411
62	H	-1.506256	2.061029	2.812706
63	C	-2.698934	3.617433	0.813082
64	H	-0.553153	3.377957	0.89891
65	H	-1.343584	2.29089	-0.232455
66	H	-3.582211	2.983488	0.661502
67	H	-2.804006	4.05693	1.81446
68	C	-2.697945	4.735958	-0.23325
69	C	-3.961425	5.60064	-0.201859
70	H	-2.583847	4.293452	-1.230367
71	H	-1.817548	5.373436	-0.08061
72	C	-3.960824	6.72091	-1.247056
73	H	-4.841343	4.962631	-0.357682
74	H	-4.077791	6.039927	0.797999
75	C	-5.22953	7.577538	-1.209053
76	H	-3.082116	7.358535	-1.090809
77	H	-3.843975	6.281254	-2.244899
78	H	-5.203321	8.368508	-1.963809
79	H	-6.120388	6.968926	-1.394913
80	H	-5.353545	8.053826	-0.231015
81	C	-0.270746	-1.037539	-1.583558
82	C	-0.866819	-1.876317	-2.716651
83	O	-0.775377	0.093221	-1.355697

84	O	0.677732	-1.553906	-0.920678
85	H	-1.363741	-1.242389	-3.452164
86	H	-0.097292	-2.488065	-3.190306
87	H	-1.614396	-2.553073	-2.288402
88	C	2.228517	1.108928	-1.440705
89	Cl	3.764876	0.681764	-2.265911
90	Cl	2.452144	2.459501	-0.254995
91	H	1.512915	1.444545	-2.18175
92	H	1.861501	0.229128	-0.918509

Table S9. The Atomic Numbering, Molecular Coordinates of [N_{1888}][FA] + DCM.

atomic numbering	atom	coordinate		
1	N	-0.061457	-0.533048	2.464483
2	C	0.227261	-0.08066	3.861626
3	H	-0.632762	0.459633	4.251602
4	H	0.427498	-0.953033	4.483616
5	H	1.096207	0.572636	3.854562
6	C	-1.32259	-1.388788	2.499775
7	C	-1.811378	-1.893996	1.148591
8	H	-2.089587	-0.780152	2.980883
9	H	-1.096549	-2.223082	3.168916
10	C	-3.140139	-2.64333	1.302774
11	H	-1.939838	-1.063765	0.451524
12	H	-1.069122	-2.551058	0.692307
13	C	-3.662913	-3.164072	-0.040164
14	H	-3.019932	-3.484889	1.998007
15	H	-3.891494	-1.978832	1.749999
16	H	-3.77176	-2.321524	-0.733143
17	H	-2.910909	-3.825979	-0.486288
18	C	-4.995088	-3.909484	0.077025
19	C	-5.516312	-4.431668	-1.265182
20	H	-5.746337	-3.244005	0.522481
21	H	-4.882101	-4.750853	0.773534
22	C	-6.85064	-5.176375	-1.154682
23	H	-5.627311	-3.59117	-1.962189
24	H	-4.766061	-5.097462	-1.710885
25	C	-7.361138	-5.693707	-2.502537
26	H	-6.738199	-6.016174	-0.457819
27	H	-7.599636	-4.509604	-0.709852
28	H	-8.313594	-6.220543	-2.396351
29	H	-7.511915	-4.87026	-3.207814
30	H	-6.644616	-6.387182	-2.954161
31	C	1.093551	-1.39398	1.937773
32	C	2.47108	-0.738474	1.959528

33	H	0.835475	-1.6317	0.903081
34	H	1.085352	-2.305166	2.541668
35	C	3.443379	-1.543593	1.082535
36	H	2.419117	0.274784	1.556807
37	H	2.867604	-0.659577	2.977787
38	C	4.80325	-0.854533	0.939948
39	H	3.577152	-2.54966	1.500262
40	H	2.993055	-1.677157	0.093664
41	H	4.647361	0.166182	0.571936
42	H	5.279998	-0.759571	1.924806
43	C	5.744356	-1.586358	-0.019955
44	C	7.072208	-0.855192	-0.234618
45	H	5.239326	-1.705993	-0.985462
46	H	5.93918	-2.599558	0.355687
47	C	8.012748	-1.572554	-1.207994
48	H	6.866205	0.155177	-0.610318
49	H	7.580266	-0.725142	0.730488
50	C	9.332702	-0.827248	-1.425261
51	H	8.218378	-2.583084	-0.833623
52	H	7.50175	-1.702153	-2.1696
53	H	9.984697	-1.358543	-2.124469
54	H	9.155383	0.174356	-1.829853
55	H	9.878396	-0.710519	-0.483162
56	C	-0.23304	0.677392	1.539876
57	C	-1.488109	1.514972	1.746806
58	H	-0.216035	0.300726	0.514244
59	H	0.65067	1.292837	1.691555
60	C	-1.482092	2.664997	0.726712
61	H	-2.38684	0.914753	1.580689
62	H	-1.54859	1.921862	2.763299
63	C	-2.741469	3.530913	0.804201
64	H	-0.595736	3.29036	0.887002
65	H	-1.383992	2.234106	-0.2746
66	H	-3.624285	2.900725	0.63553
67	H	-2.847523	3.944487	1.816427
68	C	-2.739524	4.675772	-0.213206
69	C	-4.002623	5.540022	-0.160559
70	H	-2.625118	4.258781	-1.221214
71	H	-1.858973	5.308822	-0.043872
72	C	-4.000781	6.685993	-1.177475
73	H	-4.882794	4.906593	-0.33268
74	H	-4.119147	5.954259	0.849914
75	C	-5.268899	7.542321	-1.118675
76	H	-3.121693	7.318904	-1.005006

77	H	-3.883819	6.271241	-2.185895
78	H	-5.241759	8.351788	-1.85351
79	H	-6.160098	6.939175	-1.320028
80	H	-5.393013	7.994237	-0.129149
81	C	-0.249209	-1.034992	-1.743727
82	O	-0.80808	0.055331	-1.475944
83	O	0.670682	-1.592967	-1.085332
84	H	-0.597382	-1.570946	-2.657639
85	C	2.224047	1.119319	-1.465104
86	Cl	2.427111	2.441338	-0.244344
87	Cl	3.767024	0.727445	-2.292498
88	H	1.865003	0.224106	-0.965657
89	H	1.507709	1.464734	-2.201103

Table S10. The Atomic Numbering, Molecular Coordinates of [N_{1888}][Gly] + DCM.

atomic numbering	atom	coordinate		
1	N	0.101609	-0.315481	2.685493
2	C	0.423394	0.207157	4.050623
3	H	-0.423419	0.775425	4.429483
4	H	0.629536	-0.633206	4.71336
5	H	1.297615	0.850692	3.990781
6	C	-1.160484	-1.164838	2.792894
7	C	-1.691918	-1.712681	1.475282
8	H	-1.910351	-0.536547	3.275816
9	H	-0.915323	-1.975938	3.483344
10	C	-3.000435	-2.482968	1.686267
11	H	-1.861008	-0.89981	0.766725
12	H	-0.954767	-2.366184	1.006329
13	C	-3.59214	-2.960854	0.355947
14	H	-2.828527	-3.345738	2.342822
15	H	-3.733484	-1.845533	2.198288
16	H	-3.785996	-2.086184	-0.276802
17	H	-2.845164	-3.561288	-0.17705
18	C	-4.881504	-3.770335	0.511171
19	C	-5.470436	-4.223054	-0.828408
20	H	-5.625888	-3.170366	1.051118
21	H	-4.685679	-4.650153	1.137993
22	C	-6.759687	-5.03794	-0.68502
23	H	-5.666111	-3.343021	-1.454406
24	H	-4.723904	-4.817363	-1.37036
25	C	-7.33827	-5.483142	-2.031109
26	H	-6.562183	-5.918282	-0.060878
27	H	-7.504907	-4.441753	-0.143872
28	H	-8.256881	-6.062251	-1.901621

29	H	-7.573087	-4.620424	-2.662449
30	H	-6.624403	-6.106526	-2.578602
31	C	1.243914	-1.20657	2.179727
32	C	2.619826	-0.55028	2.118965
33	H	0.957419	-1.505928	1.169562
34	H	1.255025	-2.078294	2.839224
35	C	3.5635	-1.404221	1.257129
36	H	2.552736	0.436879	1.658381
37	H	3.049301	-0.412748	3.117462
38	C	4.920441	-0.729536	1.039013
39	H	3.706604	-2.3883	1.721686
40	H	3.083069	-1.584016	0.290086
41	H	4.756104	0.269308	0.618599
42	H	5.424805	-0.581752	2.003408
43	C	5.832635	-1.517695	0.095915
44	C	7.154944	-0.804146	-0.197626
45	H	5.300087	-1.690306	-0.846482
46	H	6.036483	-2.508559	0.52288
47	C	8.066204	-1.578516	-1.15507
48	H	6.939907	0.183941	-0.624096
49	H	7.690585	-0.621153	0.743683
50	C	9.380436	-0.850769	-1.451866
51	H	8.28098	-2.566793	-0.729887
52	H	7.527726	-1.760928	-2.092823
53	H	10.011382	-1.422924	-2.138007
54	H	9.19304	0.126744	-1.907597
55	H	9.953046	-0.682365	-0.533873
56	C	-0.088542	0.84706	1.704293
57	C	-1.334113	1.701544	1.900667
58	H	-0.094391	0.418791	0.700197
59	H	0.800941	1.464846	1.801968
60	C	-1.357097	2.790782	0.816055
61	H	-2.24039	1.098246	1.799527
62	H	-1.360099	2.166891	2.893161
63	C	-2.612235	3.663686	0.882108
64	H	-0.465181	3.421094	0.913051
65	H	-1.289689	2.30251	-0.161089
66	H	-3.500816	3.027773	0.776468
67	H	-2.687094	4.134439	1.871851
68	C	-2.63983	4.748988	-0.198312
69	C	-3.900144	5.617859	-0.156996
70	H	-2.555947	4.275387	-1.184131
71	H	-1.754065	5.388303	-0.091588
72	C	-3.928055	6.704588	-1.236677

73	H	-4.785587	4.977889	-0.266596
74	H	-3.985968	6.088737	0.831472
75	C	-5.193335	7.565714	-1.188394
76	H	-3.04381	7.344174	-1.126575
77	H	-3.841675	6.233364	-2.22315
78	H	-5.187659	8.332371	-1.968201
79	H	-6.090533	6.954268	-1.328778
80	H	-5.287469	8.07298	-0.222695
81	C	-0.267569	-1.049352	-1.4389
82	N	-2.145216	-1.582656	-3.101532
83	C	-0.870826	-1.985758	-2.506494
84	O	0.727694	-1.501625	-0.799453
85	O	-0.78092	0.086622	-1.278332
86	H	-2.874938	-1.65008	-2.398306
87	H	-2.090235	-0.591186	-3.321001
88	H	-0.11854	-2.086292	-3.297088
89	H	-0.96958	-2.982176	-2.066442
90	C	2.267933	1.119959	-1.448262
91	Cl	2.500872	2.481005	-0.275813
92	Cl	3.806235	0.654997	-2.248505
93	H	1.877387	0.253012	-0.919603
94	H	1.57155	1.46314	-2.204251

Table S11. The Atomic Numbering, Molecular Coordinates of [P_{66614}][Gly] + DCM.

atomic numbering	atom	coordinate		
1	N	0.101609	-0.315481	2.685493
2	C	0.423394	0.207157	4.050623
3	H	-0.423419	0.775425	4.429483
4	H	0.629536	-0.633206	4.71336
5	H	1.297615	0.850692	3.990781
6	C	-1.160484	-1.164838	2.792894
7	C	-1.691918	-1.712681	1.475282
8	H	-1.910351	-0.536547	3.275816
9	H	-0.915323	-1.975938	3.483344
10	C	-3.000435	-2.482968	1.686267
11	H	-1.861008	-0.89981	0.766725
12	H	-0.954767	-2.366184	1.006329
13	C	-3.59214	-2.960854	0.355947
14	H	-2.828527	-3.345738	2.342822
15	H	-3.733484	-1.845533	2.198288
16	H	-3.785996	-2.086184	-0.276802
17	H	-2.845164	-3.561288	-0.17705
18	C	-4.881504	-3.770335	0.511171
19	C	-5.470436	-4.223054	-0.828408

20	P	-3.050703	1.013366	-0.921152
21	C	-1.480683	1.129262	-1.841936
22	C	-0.221612	1.415617	-1.006317
23	C	1.04692	0.958812	-1.732782
24	C	2.307255	1.178822	-0.891995
25	C	3.581999	0.667823	-1.568582
26	C	4.841052	0.86126	-0.718292
27	C	6.118088	0.351506	-1.392827
28	C	7.377069	0.542812	-0.541768
29	C	8.654357	0.035421	-1.217874
30	C	9.913596	0.226898	-0.367167
31	C	11.191092	-0.278688	-1.04437
32	C	12.450625	-0.086294	-0.194485
33	C	13.729296	-0.590906	-0.871152
34	C	14.981436	-0.392478	-0.012183
35	C	-4.312446	0.393971	-2.087115
36	C	-5.777145	0.536511	-1.64983
37	C	-6.726457	-0.115148	-2.662285
38	C	-8.199537	0.000065	-2.260639
39	C	-9.15517	-0.653242	-3.264399
40	C	-10.625346	-0.536108	-2.85306
41	C	-2.80768	-0.038295	0.550265
42	C	-3.892548	-1.080843	0.860719
43	C	-3.358812	-2.108715	1.864305
44	C	-4.36781	-3.210102	2.192924
45	C	-3.804996	-4.271597	3.144074
46	C	-4.813621	-5.37515	3.473109
47	C	-3.627296	2.664738	-0.3646
48	C	-2.63002	3.492961	0.458482
49	C	-3.277596	4.750405	1.048922
50	C	-2.293035	5.60846	1.848724
51	C	-2.931393	6.864752	2.450062
52	C	-1.938178	7.715949	3.245881
53	H	-1.618405	1.863216	-2.643099
54	H	-1.408072	0.131911	-2.286051
55	H	-0.271761	0.875644	-0.060033
56	H	-0.151845	2.4808	-0.765235
57	H	1.143439	1.485873	-2.691359
58	H	0.9382	-0.107694	-1.951289
59	H	2.181684	0.672584	0.073325
60	H	2.420219	2.246851	-0.661654
61	H	3.712861	1.17817	-2.531817
62	H	3.461417	-0.39735	-1.802583
63	H	4.710058	0.348081	0.243403

64	H	4.957936	1.926524	-0.479406
65	H	6.249337	0.865332	-2.35409
66	H	6.000623	-0.71336	-1.631837
67	H	7.246617	0.027367	0.418677
68	H	7.493314	1.607698	-0.30118
69	H	8.784593	0.55078	-2.178444
70	H	8.538175	-1.029339	-1.458269
71	H	9.784	-0.289557	0.592821
72	H	10.028987	1.291636	-0.125699
73	H	11.320116	0.237338	-2.004755
74	H	11.075952	-1.343512	-1.285198
75	H	12.322582	-0.602679	0.765968
76	H	12.566138	0.978591	0.047019
77	H	13.856613	-0.07464	-1.830601
78	H	13.613606	-1.654927	-1.111273
79	H	15.878701	-0.760658	-0.517624
80	H	14.893052	-0.924908	0.940311
81	H	15.137929	0.666852	0.215701
82	H	-4.137689	0.941199	-3.020724
83	H	-4.030264	-0.646193	-2.266506
84	H	-5.927317	0.069208	-0.67249
85	H	-6.043596	1.59344	-1.536581
86	H	-6.578552	0.345104	-3.647477
87	H	-6.457613	-1.171926	-2.774704
88	H	-8.343055	-0.459602	-1.274127
89	H	-8.465258	1.059295	-2.145291
90	H	-9.011253	-0.194202	-4.250037
91	H	-8.888245	-1.710566	-3.378644
92	H	-11.284963	-1.009904	-3.585027
93	H	-10.802341	-1.016062	-1.885314
94	H	-10.926662	0.512448	-2.762173
95	H	-1.866408	-0.553548	0.345924
96	H	-2.640214	0.624905	1.403201
97	H	-4.794636	-0.603018	1.26063
98	H	-4.162632	-1.604249	-0.058516
99	H	-2.453075	-2.561231	1.448813
100	H	-3.045917	-1.60419	2.786528
101	H	-5.270393	-2.766637	2.634965
102	H	-4.688528	-3.694358	1.261337
103	H	-2.908335	-4.713735	2.694677
104	H	-3.472013	-3.786271	4.069353
105	H	-4.386324	-6.120011	4.150172
106	H	-5.707622	-4.962444	3.952273
107	H	-5.135389	-5.896154	2.565681

108	H	-4.535317	2.477608	0.219199
109	H	-3.938596	3.217531	-1.257347
110	H	-1.786834	3.784607	-0.173332
111	H	-2.209871	2.889771	1.27017
112	H	-4.114828	4.457617	1.695172
113	H	-3.710976	5.351004	0.238976
114	H	-1.457445	5.901909	1.200328
115	H	-1.855562	5.003852	2.653426

Table S12. The Atomic Numbering, Molecular Coordinates of [N_{1888}][Ac].

atomic numbering	atom	coordinate		
1	N	0.748113	-0.067382	-2.321999
2	C	1.070483	-0.688416	-3.643865
3	H	0.163324	-1.11064	-4.072405
4	H	1.472295	0.075005	-4.30999
5	H	1.807042	-1.47536	-3.502576
6	C	-0.306888	1.010628	-2.557283
7	C	-0.746807	1.766865	-1.308459
8	H	-1.153528	0.504465	-3.021928
9	H	0.121067	1.685352	-3.303392
10	C	-2.083935	2.480086	-1.538856
11	H	-0.846836	1.089005	-0.455379
12	H	0.013977	2.498437	-1.02536
13	C	-2.539451	3.242911	-0.290679
14	H	-2.015341	3.171316	-2.390331
15	H	-2.849322	1.73834	-1.803933
16	H	-2.565497	2.547695	0.556067
17	H	-1.788114	4.001863	-0.038537
18	C	-3.905405	3.914344	-0.45477
19	C	-4.356357	4.680009	0.792733
20	H	-4.656205	3.151944	-0.700957
21	H	-3.876487	4.601574	-1.311104
22	C	-5.728345	5.344543	0.640625
23	H	-4.378813	3.993729	1.648743
24	H	-3.608883	5.445815	1.037974
25	C	-6.167594	6.106363	1.894361
26	H	-5.704638	6.029685	-0.215978
27	H	-6.474387	4.578081	0.396985
28	H	-7.14921	6.569818	1.760598
29	H	-6.229075	5.43744	2.75864
30	H	-5.454902	6.899612	2.14203
31	C	1.985681	0.606113	-1.723527
32	C	3.180674	-0.301268	-1.459782
33	H	1.659019	1.008471	-0.761942

34	H	2.24989	1.411003	-2.415192
35	C	4.24033	0.4826	-0.671135
36	H	2.873634	-1.15401	-0.850574
37	H	3.6181	-0.687828	-2.387703
38	C	5.473118	-0.362688	-0.340659
39	H	4.549228	1.36709	-1.244172
40	H	3.776011	0.841097	0.253038
41	H	5.158476	-1.246065	0.229245
42	H	5.926075	-0.739145	-1.267947
43	C	6.527547	0.404125	0.462994
44	C	7.757015	-0.438872	0.813931
45	H	6.069856	0.782054	1.385356
46	H	6.844625	1.28857	-0.105087
47	C	8.812619	0.325563	1.619388
48	H	7.439485	-1.322519	1.382791
49	H	8.213521	-0.819788	-0.109493
50	C	10.035858	-0.52756	1.966987
51	H	9.13003	1.208382	1.050828
52	H	8.355659	0.705343	2.54113
53	H	10.772585	0.040982	2.541469
54	H	9.749654	-1.400072	2.56302
55	H	10.529966	-0.894393	1.061397
56	C	0.248908	-1.142639	-1.34472
57	C	-1.178735	-1.627092	-1.552845
58	H	0.340291	-0.715312	-0.346089
59	H	0.948687	-1.972308	-1.443866
60	C	-1.493795	-2.709717	-0.510483
61	H	-1.880031	-0.804169	-1.398517
62	H	-1.340001	-2.021639	-2.564034
63	C	-2.942769	-3.196951	-0.588905
64	H	-0.813908	-3.56169	-0.643485
65	H	-1.300041	-2.285513	0.47978
66	H	-3.614474	-2.340391	-0.451314
67	H	-3.14954	-3.594482	-1.592118
68	C	-3.271968	-4.265518	0.457598
69	C	-4.725754	-4.743816	0.402423
70	H	-3.057854	-3.865404	1.456064
71	H	-2.602237	-5.125035	0.32241
72	C	-5.058142	-5.811529	1.449752
73	H	-5.394679	-3.884106	0.539208
74	H	-4.941473	-5.140646	-0.598735
75	C	-6.514762	-6.279927	1.387673
76	H	-4.389913	-6.6707	1.313017
77	H	-4.84226	-5.41406	2.448762

78	H	-6.725365	-7.040594	2.144729
79	H	-7.203163	-5.445024	1.55359
80	H	-6.748292	-6.710342	0.408358
81	C	0.316162	0.453673	1.863411
82	C	0.340021	0.799476	3.357092
83	O	-0.7559	-0.012168	1.388718
84	O	1.368626	0.686177	1.201729
85	H	-0.398274	0.21247	3.90564
86	H	1.338223	0.649246	3.772766
87	H	0.087182	1.859125	3.470923

Table S13. The Atomic Numbering, Molecular Coordinates of [N_{1888}][FA].

atomic numbering	atom	coordinate		
1	N	-0.906834	0.042865	1.223801
2	C	-1.157216	-0.586332	2.574547
3	H	-0.187902	-0.838343	3.025924
4	H	-1.678194	0.14421	3.194025
5	H	-1.776856	-1.471694	2.445654
6	C	0.154095	1.130963	1.442351
7	C	0.684524	1.819785	0.192407
8	H	0.990542	0.657641	1.964232
9	H	-0.313285	1.851807	2.117551
10	C	1.765053	2.833425	0.601643
11	H	1.142997	1.084026	-0.474651
12	H	-0.103596	2.330046	-0.373161
13	C	2.428912	3.504085	-0.603606
14	H	1.317608	3.602056	1.245327
15	H	2.516001	2.311171	1.202399
16	H	2.881105	2.732037	-1.239319
17	H	1.668463	4.003683	-1.219616
18	C	3.504744	4.518752	-0.205161
19	C	4.192905	5.178767	-1.403531
20	H	4.256227	4.017599	0.416762
21	H	3.054175	5.295109	0.426957
22	C	5.268044	6.195711	-1.006789
23	H	4.646317	4.402259	-2.03354
24	H	3.439148	5.675844	-2.02897
25	C	5.951989	6.846911	-2.212241
26	H	4.81493	6.971905	-0.377893
27	H	6.019682	5.698445	-0.382058
28	H	6.713789	7.567532	-1.901697
29	H	6.440868	6.094997	-2.840061
30	H	5.226255	7.377313	-2.837431
31	C	-2.172851	0.692375	0.693276

32	C	-3.396814	-0.20669	0.540324
33	H	-1.914567	1.135556	-0.268458
34	H	-2.397024	1.508018	1.383992
35	C	-4.59477	0.604588	0.026557
36	H	-3.194514	-1.02159	-0.15995
37	H	-3.659788	-0.662856	1.498253
38	C	-5.857193	-0.24599	-0.144602
39	H	-4.80405	1.427706	0.721042
40	H	-4.338129	1.070017	-0.933242
41	H	-5.647689	-1.070464	-0.837803
42	H	-6.113163	-0.711516	0.815492
43	C	-7.058986	0.553629	-0.656622
44	C	-8.323375	-0.293865	-0.826511
45	H	-6.802424	1.018687	-1.617155
46	H	-7.266583	1.379409	0.035954
47	C	-9.527437	0.50298	-1.339119
48	H	-8.116097	-1.120576	-1.518443
49	H	-8.580342	-0.758878	0.134134
50	C	-10.785682	-0.353867	-1.503816
51	H	-9.733429	1.328979	-0.647518
52	H	-9.270184	0.966439	-2.29936
53	H	-11.628723	0.238096	-1.87025
54	H	-10.616474	-1.1688	-2.214809
55	H	-11.083043	-0.803283	-0.551017
56	C	-0.427341	-1.016524	0.237588
57	C	0.904541	-1.672677	0.572392
58	H	-0.386703	-0.526305	-0.737007
59	H	-1.217209	-1.767531	0.197742
60	C	1.277535	-2.696742	-0.505565
61	H	1.696965	-0.928117	0.666991
62	H	0.856096	-2.156871	1.549626
63	C	2.613297	-3.383804	-0.202602
64	H	0.490065	-3.45782	-0.595443
65	H	1.34015	-2.202105	-1.484689
66	H	3.392305	-2.620077	-0.096748
67	H	2.546959	-3.881346	0.772229
68	C	3.027238	-4.400073	-1.270169
69	C	4.359198	-5.091525	-0.964001
70	H	3.09667	-3.897254	-2.244021
71	H	2.241304	-5.159998	-1.375352
72	C	4.78073	-6.107172	-2.030726
73	H	5.144431	-4.332402	-0.855991
74	H	4.290238	-5.595565	0.008607
75	C	6.113019	-6.792129	-1.712896

76	H	3.994712	-6.86509	-2.137632
77	H	4.85047	-5.60176	-3.001956
78	H	6.391241	-7.511031	-2.488802
79	H	6.9218	-6.058994	-1.631346
80	H	6.060366	-7.331203	-0.761597
81	C	2.720979	-0.59251	3.317198
82	O	1.622126	-1.095651	3.669434
83	O	2.935361	0.16184	2.335215
84	H	3.605191	-0.851114	3.948335

Table S14. The Atomic Numbering, Molecular Coordinates of [N_{1888}][Gly].

atomic numbering	atom	coordinate		
1	N	-1.113575	0.039022	0.996888
2	C	-1.253627	-0.686522	2.314671
3	H	-0.249224	-0.927323	2.688209
4	H	-1.760913	-0.020017	3.012738
5	H	-1.847038	-1.586456	2.164877
6	C	-0.082625	1.154175	1.224854
7	C	0.346605	1.940944	-0.006053
8	H	0.80201	0.685699	1.667287
9	H	-0.536984	1.811979	1.969771
10	C	1.413595	2.967661	0.406085
11	H	0.789813	1.267599	-0.745426
12	H	-0.49161	2.454902	-0.490478
13	C	1.976747	3.743195	-0.787425
14	H	0.980182	3.673386	1.126816
15	H	2.218499	2.436765	0.923483
16	H	2.413761	3.033931	-1.501997
17	H	1.162406	4.254055	-1.31952
18	C	3.040613	4.768709	-0.384584
19	C	3.628616	5.535318	-1.572781
20	H	3.846751	4.255256	0.153245
21	H	2.605053	5.482036	0.327286
22	C	4.692691	6.561893	-1.171025
23	H	4.066335	4.821798	-2.283217
24	H	2.820426	6.045832	-2.113675
25	C	5.276468	7.320136	-2.366513
26	H	4.255277	7.275198	-0.461683
27	H	5.498659	6.051012	-0.630706
28	H	6.032478	8.04531	-2.052508
29	H	5.748662	6.632318	-3.075345
30	H	4.495273	7.865267	-2.906352
31	C	-2.434439	0.668579	0.590062
32	C	-3.628539	-0.270271	0.44005

33	H	-2.252536	1.191519	-0.348829
34	H	-2.648664	1.42123	1.351791
35	C	-4.888962	0.523865	0.068506
36	H	-3.439315	-1.017161	-0.33562
37	H	-3.810447	-0.812163	1.371842
38	C	-6.122664	-0.367901	-0.102501
39	H	-5.089239	1.275183	0.842447
40	H	-4.711783	1.079525	-0.860768
41	H	-5.922331	-1.119111	-0.876911
42	H	-6.298284	-0.925511	0.826075
43	C	-7.386708	0.414759	-0.470644
44	C	-8.621575	-0.474483	-0.644309
45	H	-7.209516	0.974148	-1.398227
46	H	-7.58679	1.165574	0.304527
47	C	-9.887971	0.305576	-1.012102
48	H	-8.421879	-1.225572	-1.419652
49	H	-8.798792	-1.034711	0.282969
50	C	-11.115667	-0.593236	-1.183512
51	H	-10.086971	1.055348	-0.236705
52	H	-9.709983	0.865066	-1.938561
53	H	-12.004279	-0.012469	-1.445175
54	H	-10.95508	-1.331867	-1.975269
55	H	-11.334664	-1.140246	-0.260996
56	C	-0.658914	-0.933109	-0.086672
57	C	0.714009	-1.553567	0.125434
58	H	-0.696474	-0.378402	-1.026138
59	H	-1.421831	-1.71124	-0.130465
60	C	1.070246	-2.485808	-1.037653
61	H	1.479826	-0.782953	0.228414
62	H	0.739579	-2.105371	1.066921
63	C	2.451257	-3.123098	-0.849152
64	H	0.314632	-3.27726	-1.136748
65	H	1.056497	-1.926627	-1.98325
66	H	3.198476	-2.329084	-0.735814
67	H	2.461904	-3.681772	0.094418
68	C	2.854259	-4.051003	-1.998099
69	C	4.233553	-4.688443	-1.805254
70	H	2.844664	-3.488224	-2.941051
71	H	2.101757	-4.843251	-2.108642
72	C	4.643929	-5.617256	-2.952435
73	H	4.985508	-3.896914	-1.693285
74	H	4.244166	-5.25066	-0.862672
75	C	6.024369	-6.247921	-2.748055
76	H	3.891629	-6.408198	-3.062648

77	H	4.63352	-5.053766	-3.893718
78	H	6.293374	-6.905618	-3.579497
79	H	6.799143	-5.479006	-2.666203
80	H	6.052516	-6.842341	-1.829307
81	C	2.675066	-0.6549	2.768621
82	N	3.929476	-2.007387	4.552213
83	C	4.009628	-1.168968	3.352881
84	O	2.75976	0.230796	1.876649
85	O	1.610338	-1.187441	3.190527
86	H	3.667982	-1.42348	5.34171
87	H	3.138763	-2.636419	4.431731
88	H	4.497558	-1.736455	2.550703
89	H	4.655678	-0.308516	3.544323

Table S15. The Atomic Numbering, Molecular Coordinates of [P_{66614}][Gly].

atomic numbering	atom	coordinate		
1	P	-3.134399	-1.430174	-0.179927
2	C	-1.518638	-2.278034	-0.364256
3	C	-0.317142	-1.522892	0.226674
4	C	1.01521	-2.144836	-0.199155
5	C	2.20661	-1.353785	0.351069
6	C	3.558842	-1.894126	-0.120731
7	C	4.750331	-1.106092	0.43183
8	C	6.105618	-1.629679	-0.052634
9	C	7.298555	-0.846802	0.504157
10	C	8.653153	-1.368553	0.015362
11	C	9.847163	-0.588383	0.573685
12	C	11.201231	-1.110816	0.08396
13	C	12.395779	-0.331939	0.64266
14	C	13.750621	-0.85364	0.153021
15	C	14.936857	-0.067512	0.718696
16	C	-4.390511	-2.759402	-0.024334
17	C	-5.859824	-2.34767	-0.199828
18	C	-6.812919	-3.531246	0.003123
19	C	-8.284618	-3.149575	-0.181081
20	C	-9.246508	-4.324421	0.024271
21	C	-10.714664	-3.932392	-0.162785
22	C	-3.090137	-0.338014	1.264288
23	C	-4.30121	0.588093	1.46148
24	C	-4.019759	1.61293	2.567715
25	C	-5.160229	2.617905	2.745828
26	C	-4.884002	3.652752	3.841536
27	C	-6.018432	4.667727	4.006063
28	C	-3.49704	-0.467685	-1.686519

29	C	-2.528495	0.704292	-1.895608
30	C	-2.803877	1.477762	-3.186572
31	C	-1.884723	2.698492	-3.310536
32	C	-2.061658	3.466485	-4.622668
33	C	-1.149233	4.693136	-4.715958
34	H	-1.386861	-2.429949	-1.441598
35	H	-1.625597	-3.272198	0.083918
36	H	-0.392273	-1.521768	1.319563
37	H	-0.313889	-0.475114	-0.089938
38	H	1.06923	-2.149867	-1.295792
39	H	1.075773	-3.194647	0.121513
40	H	2.172701	-1.36721	1.44863
41	H	2.092681	-0.307306	0.048406
42	H	3.59176	-1.87257	-1.217946
43	H	3.657851	-2.949875	0.167294
44	H	4.723348	-1.131089	1.52914
45	H	4.644936	-0.051364	0.149363
46	H	6.133526	-1.598994	-1.149713
47	H	6.206491	-2.687607	0.224177
48	H	7.272497	-0.878928	1.601237
49	H	7.196866	0.210935	0.229347
50	H	8.679539	-1.334193	-1.081626
51	H	8.753372	-2.427381	0.288157
52	H	9.821077	-0.622705	1.670663
53	H	9.747229	0.470204	0.301025
54	H	11.227337	-1.075638	-1.013016
55	H	11.300532	-2.169867	0.355956
56	H	12.370544	-0.36693	1.73981
57	H	12.297188	0.727283	0.371046
58	H	13.775426	-0.817481	-0.943046
59	H	13.84844	-1.911934	0.424731
60	H	15.890469	-0.459628	0.353866
61	H	14.953515	-0.114107	1.812379
62	H	14.879808	0.988263	0.434799
63	H	-4.121518	-3.52178	-0.764138
64	H	-4.23021	-3.211121	0.960958
65	H	-6.116437	-1.552879	0.505935
66	H	-6.010342	-1.933911	-1.202208
67	H	-6.552614	-4.332489	-0.700065
68	H	-6.666879	-3.945818	1.008451
69	H	-8.541374	-2.344894	0.519776
70	H	-8.429314	-2.73515	-1.187111
71	H	-8.988364	-5.128093	-0.675953
72	H	-9.101374	-4.73714	1.029935

73	H	-11.379048	-4.78705	-0.01075
74	H	-11.00725	-3.152247	0.546938
75	H	-10.893613	-3.546228	-1.171171
76	H	-2.918281	-0.970485	2.142069
77	H	-2.196783	0.298048	1.114097
78	H	-4.502934	1.129622	0.53105
79	H	-5.206378	0.020111	1.703866
80	H	-3.846237	1.084112	3.514179
81	H	-3.090634	2.135215	2.320316
82	H	-5.330885	3.140457	1.79528
83	H	-6.09487	2.08768	2.976102
84	H	-4.713872	3.133536	4.792701
85	H	-3.950234	4.177483	3.609331
86	H	-5.795968	5.393671	4.792953
87	H	-6.185051	5.223987	3.078082
88	H	-6.958426	4.170219	4.26699
89	H	-4.529076	-0.112946	-1.604327
90	H	-3.466477	-1.176381	-2.522848
91	H	-1.491675	0.360755	-1.896568
92	H	-2.588212	1.381562	-1.040715
93	H	-3.853299	1.802883	-3.214274
94	H	-2.66163	0.8187	-4.053862
95	H	-0.843233	2.377129	-3.199561
96	H	-2.074463	3.372055	-2.465737
97	H	-3.108952	3.778777	-4.725378
98	H	-1.859677	2.79405	-5.466103
99	H	-1.286252	5.226298	-5.661173
100	H	-0.096566	4.40359	-4.642291
101	H	-1.352895	5.395799	-3.901956
102	C	-0.229311	2.212231	0.054314
103	N	-0.205341	4.537119	1.149284
104	C	0.290468	3.66843	0.077129
105	O	0.337364	1.430257	-0.745739
106	O	-1.214443	1.939763	0.803715
107	H	-1.196009	4.337616	1.267924
108	H	0.216048	4.244793	2.026595
109	H	1.382843	3.643082	0.097364
110	H	0.019836	4.105193	-0.892108

Table S16. The Atomic Numbering, Molecular Coordinates of DCM.

atomic numbering	atom	coordinate		
1	C	-0.000001	0.769422	0
2	Cl	1.498075	-0.216788	0
3	Cl	-1.498075	-0.216788	0

4	H	0	1.377125	0.899144
5	H	0	1.377146	-0.899131

Table S17. The Atomic Numbering, Molecular Coordinates of $[N_{1888}]^{+}$-DCM.

atomic numbering	atom	coordinate		
1	N	0.848133	0.170613	2.167365
2	C	1.199486	0.827849	3.473612
3	H	0.29369	1.211124	3.936787
4	H	1.660054	0.088664	4.12759
5	H	1.892098	1.64581	3.294627
6	C	-0.195901	-0.909723	2.463425
7	C	-0.719961	-1.670196	1.252488
8	H	-1.013406	-0.404374	2.977175
9	H	0.274056	-1.588439	3.178237
10	C	-1.877326	-2.600484	1.641917
11	H	-1.07554	-0.970277	0.495653
12	H	0.073517	-2.263387	0.79221
13	C	-2.462576	-3.320301	0.421637
14	H	-1.532536	-3.33931	2.37481
15	H	-2.668553	-2.020716	2.132317
16	H	-2.830501	-2.569948	-0.290374
17	H	-1.663899	-3.875148	-0.088517
18	C	-3.601787	-4.282754	0.768558
19	C	-4.190367	-4.983994	-0.459508
20	H	-4.395739	-3.731509	1.28767
21	H	-3.236965	-5.035711	1.478039
22	C	-5.331539	-5.948954	-0.121205
23	H	-4.554957	-4.228987	-1.16857
24	H	-3.395153	-5.534063	-0.979731
25	C	-5.91148	-6.642834	-1.35641
26	H	-4.967233	-6.702592	0.587105
27	H	-6.125198	-5.399239	0.398791
28	H	-6.722156	-7.324233	-1.087026
29	H	-6.312656	-5.913148	-2.066762
30	H	-5.145665	-7.22646	-1.876807
31	C	2.090353	-0.500604	1.574059
32	C	3.271224	0.408442	1.251219
33	H	1.753992	-0.998711	0.667337
34	H	2.383965	-1.266664	2.29528
35	C	4.415159	-0.421132	0.645162
36	H	2.980186	1.180612	0.534421
37	H	3.637745	0.914914	2.148432
38	C	5.63596	0.432766	0.287412
39	H	4.713827	-1.205252	1.351406

40	H	4.055131	-0.935305	-0.254326
41	H	5.335127	1.218404	-0.416908
42	H	5.995473	0.947405	1.187201
43	C	6.779951	-0.383943	-0.321592
44	C	8.00234	0.465498	-0.682784
45	H	6.418584	-0.900093	-1.22012
46	H	7.079652	-1.169991	0.383056
47	C	9.148833	-0.348356	-1.292062
48	H	7.702867	1.251933	-1.387559
49	H	8.363666	0.982629	0.215455
50	C	10.365079	0.50991	-1.649688
51	H	9.447405	-1.133581	-0.587069
52	H	8.786733	-0.864913	-2.189213
53	H	11.16668	-0.094362	-2.081686
54	H	10.101404	1.283035	-2.378108
55	H	10.767055	1.012158	-0.764274
56	C	0.317259	1.218592	1.183715
57	C	-1.068013	1.779363	1.482244
58	H	0.332801	0.752168	0.199763
59	H	1.054226	2.020625	1.183391
60	C	-1.373907	2.944651	0.527677
61	H	-1.829466	1.006141	1.351739
62	H	-1.140586	2.135303	2.514498
63	C	-2.818126	3.439964	0.648879
64	H	-0.682939	3.771738	0.729697
65	H	-1.185577	2.628197	-0.504186
66	H	-3.503239	2.60935	0.435959
67	H	-3.015853	3.741975	1.685072
68	C	-3.131383	4.608285	-0.290736
69	C	-4.57993	5.094995	-0.18688
70	H	-2.923029	4.306515	-1.325134
71	H	-2.451547	5.441562	-0.072806
72	C	-4.897334	6.263391	-1.126012
73	H	-5.259748	4.261086	-0.404994
74	H	-4.788979	5.395735	0.847861
75	C	-6.347655	6.740878	-1.01499
76	H	-4.218276	7.096182	-0.906906
77	H	-4.68738	5.962015	-2.159426
78	H	-6.546765	7.573563	-1.694197
79	H	-7.047501	5.936068	-1.260808
80	H	-6.574681	7.078512	0.001014
81	C	-0.905305	-1.159975	-2.525013
82	Cl	0.823059	-0.956751	-2.059055
83	Cl	-1.96324	0.127838	-1.860294

84	H	-0.959768	-1.11493	-3.607489
85	H	-1.246598	-2.112851	-2.132973

Table S18. The Atomic Numbering, Molecular Coordinates of $[P_{66614}]^{+}$-DCM.

atomic numbering	atom	coordinate		
1	P	2.798153	-0.096803	-0.748972
2	C	1.139657	-0.739275	-1.161816
3	C	-0.0768	0.060634	-0.674211
4	C	-1.388645	-0.605535	-1.106082
5	C	-2.626204	0.155524	-0.62175
6	C	-3.942557	-0.504098	-1.044201
7	C	-5.181903	0.257841	-0.564983
8	C	-6.499625	-0.399966	-0.985697
9	C	-7.739211	0.362917	-0.508651
10	C	-9.057633	-0.293562	-0.929223
11	C	-10.297257	0.470001	-0.453388
12	C	-11.616106	-0.185265	-0.874645
13	C	-12.855692	0.57858	-0.399527
14	C	-14.175875	-0.075308	-0.820373
15	C	-15.407373	0.697366	-0.339768
16	C	3.982766	-1.11982	-1.703309
17	C	5.400169	-1.259234	-1.123779
18	C	6.310121	-2.070343	-2.053871
19	C	7.722636	-2.251568	-1.489824
20	C	8.641379	-3.060517	-2.411817
21	C	10.050337	-3.236624	-1.839852
22	C	2.899589	1.646524	-1.274643
23	C	4.272671	2.310099	-1.079568
24	C	4.249695	3.788803	-1.484888
25	C	5.608192	4.474129	-1.309329
26	C	5.593272	5.953286	-1.710064
27	C	6.954555	6.630494	-1.532328
28	C	3.197243	-0.215027	1.026723
29	C	2.392054	0.687353	1.973645
30	C	2.848678	0.511771	3.426614
31	C	2.05209	1.378167	4.406333
32	C	2.496627	1.20675	5.862797
33	C	1.692581	2.073455	6.835217
34	H	1.111957	-1.761017	-0.774448
35	H	1.129798	-0.816657	-2.255555
36	H	-0.04173	1.082366	-1.066469
37	H	-0.062005	0.139796	0.41519
38	H	-1.414303	-1.630516	-0.716791
39	H	-1.413162	-0.68798	-2.199662

40	H	-2.595235	1.182717	-1.006678
41	H	-2.59617	0.23718	0.472334
42	H	-3.974524	-1.530221	-0.656183
43	H	-3.970131	-0.59024	-2.137841
44	H	-5.148925	1.283801	-0.953496
45	H	-5.153419	0.345222	0.528807
46	H	-6.533245	-1.425735	-0.596418
47	H	-6.527343	-0.488523	-2.079354
48	H	-7.70514	1.388684	-0.897882
49	H	-7.711456	0.451708	0.585108
50	H	-9.092227	-1.319232	-0.539621
51	H	-9.085008	-0.382887	-2.022916
52	H	-10.262159	1.49581	-0.842551
53	H	-10.270217	0.559013	0.640395
54	H	-11.651496	-1.211047	-0.485282
55	H	-11.642928	-0.274489	-1.968422
56	H	-12.820865	1.604637	-0.788636
57	H	-12.829678	0.668021	0.694442
58	H	-14.210257	-1.100169	-0.43097
59	H	-14.201372	-0.163908	-1.913363
60	H	-16.334622	0.210101	-0.653194
61	H	-15.414041	1.715841	-0.740991
62	H	-15.423113	0.77268	0.75221
63	H	3.518567	-2.106192	-1.798631
64	H	4.013477	-0.688523	-2.709855
65	H	5.84442	-0.273554	-0.95317
66	H	5.349348	-1.754392	-0.14893
67	H	5.85936	-3.054067	-2.234282
68	H	6.36679	-1.574043	-3.030489
69	H	8.170349	-1.266335	-1.306517
70	H	7.662963	-2.747568	-0.512598
71	H	8.192512	-4.044109	-2.594532
72	H	8.699607	-2.564385	-3.387934
73	H	10.683757	-3.815857	-2.515798
74	H	10.534373	-2.268587	-1.67803
75	H	10.023583	-3.759603	-0.878945
76	H	2.600345	1.67213	-2.328027
77	H	2.128967	2.186197	-0.716035
78	H	4.582955	2.228741	-0.031961
79	H	5.027879	1.785409	-1.673639
80	H	3.930044	3.872392	-2.53076
81	H	3.494235	4.315188	-0.888636
82	H	5.92798	4.385884	-0.263026
83	H	6.362386	3.945189	-1.906017

84	H	5.272584	6.039928	-2.754997
85	H	4.839128	6.480321	-1.113544
86	H	6.915721	7.682417	-1.824791
87	H	7.283938	6.587022	-0.489639
88	H	7.720463	6.143353	-2.143564
89	H	4.263917	0.015739	1.104441
90	H	3.081842	-1.266686	1.298486
91	H	1.327998	0.4511	1.90583
92	H	2.502386	1.737447	1.682156
93	H	3.915778	0.753833	3.505418
94	H	2.748422	-0.54358	3.707416
95	H	0.985507	1.13294	4.322512
96	H	2.146985	2.433603	4.12056
97	H	3.562171	1.451954	5.945494
98	H	2.402416	0.151494	6.145793
99	H	2.029207	1.932248	7.864986
100	H	0.627372	1.825643	6.796258
101	H	1.795481	3.135947	6.594327
102	C	1.237811	-4.05113	1.669326
103	Cl	0.4712	-2.45971	1.997933
104	Cl	2.59264	-3.942713	0.48575
105	H	1.642892	-4.429563	2.601843
106	H	0.483875	-4.709324	1.250617

Table S19. The Atomic Numbering, Molecular Coordinates of $[Ac]^{-}$-DCM.

atomic numbering	atom	coordinate		
1	C	2.296896	-0.00003	0.012339
2	C	3.847753	0.000025	-0.03537
3	O	1.762738	-0.000028	1.150712
4	O	1.724147	-0.000042	-1.110378
5	H	4.279369	-0.000283	0.968186
6	H	4.192918	-0.880957	-0.586429
7	H	4.192837	0.881428	-0.585813
8	C	-1.046351	-0.000001	0.054496
9	Cl	-2.068203	1.499172	-0.015003
10	Cl	-2.068241	-1.499147	-0.015
11	H	-0.346133	-0.000011	-0.780414
12	H	-0.484306	-0.000007	0.983064

Table S20. The Atomic Numbering, Molecular Coordinates of $[FA]^{-}$-DCM.

atomic numbering	atom	coordinate		
1	C	2.792949	0.000012	-0.000002
2	O	2.256884	0.000045	1.134987
3	O	2.256875	-0.000043	-1.134986

4	H	3.92223	0.000043	-0.000006
5	C	-0.553534	0.000006	-0.000001
6	Cl	-1.577258	1.499165	-0.000015
7	Cl	-1.57723	-1.499176	0.000017
8	H	0.078756	-0.000001	-0.884308
9	H	0.078758	0.000021	0.884305

Table S21. The Atomic Numbering, Molecular Coordinates of $[Gly]^{-}$-DCM.

atomic numbering	atom	coordinate		
1	C	1.835345	0.079758	0.294216
2	N	4.189873	-0.154305	-0.722734
3	C	3.381567	0.151177	0.471285
4	O	1.170686	0.116722	1.360755
5	O	1.407882	0.023161	-0.888542
6	H	4.123538	-1.155235	-0.891912
7	H	3.68112	0.248456	-1.509501
8	H	3.613526	1.169405	0.80645
9	H	3.668609	-0.51248	1.291767
10	C	-1.497281	-0.003255	-0.048513
11	Cl	-2.474123	-1.531468	-0.008037
12	Cl	-2.562915	1.461684	-0.14779
13	H	-0.884448	0.056282	0.84957
14	H	-0.848141	-0.025109	-0.917802
